# Description of *Saprolegnia velencensis* sp. n. (Oomycota), a novel water mold species from Lake Velence, Hungary

**DOI:** 10.1371/journal.pone.0298814

**Published:** 2024-03-20

**Authors:** Viktória Verebélyi, Noémi Erdei, Tímea Hardy, Edit Eszterbauer

**Affiliations:** HUN-REN Veterinary Medical Research Institute, Budapest, Hungary; ICAR - Directorate of Coldwater Fisheries Research, INDIA

## Abstract

Here, we describe a novel water mold species, *Saprolegnia velencensis* sp. n. from Lake Velence, in Hungary. Two strains (SAP239 and SAP241) were isolated from lake water, and characterized using morphological and molecular markers. In addition, phylogenetic analyses based on ITS–rDNA regions and on the RNA polymerase II B subunit (RPB2) gene complemented the study. The ITS–rDNA of the two strains was 100% identical, showed the highest similarity to that of *S*. *ferax* (with 94.4% identity), and they formed a separate cluster in both the ITS–rDNA and RPB2-based maximum likelihood phylogenetic trees with high bootstrap support. Although mature oogonia and antheridia were not seen under *in vitro* conditions, the *S*. *velencensis* sp. n. could be clearly distinguished from its closest relative, *S*. *ferax*, by the length and width of sporangia, as the new species had shorter and narrower sporangia (163.33±70.07 and 36.69±8.27 μm, respectively) than those of *S*. *ferax*. The two species also differed in the size of the secondary cysts (11.63±1.77 μm), which were slightly smaller in *S*. *ferax*. Our results showed that *S*. *velencensis* sp. n. could not be identified with any of the previously described water mold species, justifying its description as a new species.

## Introduction

*Saprolegnia* spp. water molds belong to the fungal-like organisms, the oomycetes (Oomycota). The genus *Saprolegnia* contains important pathogens that cause severe losses in aquaculture by damaging both fish eggs and adult fish, however, they are also hazardous to other species in natural water ecosystems [1]. Saprolegniosis, the disease caused by *Saprolegnia* spp. shows symptoms visible to the naked eye, when whitish, cotton-like patches of hyphae grow on fish skin or on the surface of eggs [2–5].

The last survey of water mold species in Hungary was published in the 1970s [6], thus there is no up-to-date information on the current species distribution of water mold species in Hungary, although this would be relevant for the development of effective protection against saprolegniosis. In a recent study, Pavić et al. [2] have concluded that *Saprolegnia parasitica* was the dominant species in trout farms in Croatia, whereas *S*. *australis* and *S*. *ferax* were more abundant in natural waters. They have emphasized the importance of studying natural waters associated with fish farms to gain a global picture of the epidemiology of water molds. Similar conclusions have been drawn by Nam et al. [7], who studied the diversity and distribution of oomycetes in freshwater ecosystems in South Korea. They have detected dozens of water mold species and found that *S*. *ferax* was the most abundant one in the natural waters studied. Also, high species diversity of *Saprolegnia* spp.–including the newly described *S*. *atlantica*–has recently been found in freshwater habitats in the Atlantic Rainforest of Brazil [8].

The species-level identification of *Saprolegnia* spp. is traditionally based on morphology [9,10]. However, it is often rather challenging, as vegetative and reproductive structures can be ambivalent and highly variable within a species or similar between species [4,11–13]. Moreover, some species such as *S*. *parasitica*, often fail to produce sexual reproductive structures (i.e. oogonia and antheridia) *in vitro*, which would be necessary for the morphology-based identification [5,10,14]. Some studies have suggested that changing favorable and unfavorable conditions can induce the production of sexual reproductive structures of *Saprolegnia* strains *in vitro*, however it could not be applied successfully in every case [11,15–17].

The DNA sequence-based, molecular identification is a more effective tool with a great opportunity to clarify *Saprolegnia* phylogeny. The internal transcribed spacer region (ITS–rDNA) is the most frequently used molecular marker for the identification of *Saprolegnia* spp. Sandoval-Sierra et al. [4] revised the NCBI GenBank entries of *Saprolegnia* spp. and proposed the use of molecular operational taxonomic units including “reference” DNA sequences (MOTUs; i.e. highly supported phylogenetic clusters) for the phylogenetic analysis to avoid false identification. In their study, 961 *Saprolegnia* ITS–rDNA sequences were analysed, resulting in the determination of 29 DNA-based MOTUs. Of them, 18 MOTUs were previously described, whereas 11 could potentially be new species that have not been described yet. Several studies have focused on other genetic markers, which may provide a more detailed picture of the intraspecific diversity, such as cytochrome oxidase (COX I and COX II), or DNA-directed RNA polymerase II subunit (RPB2) gene [18–20]. Sarowar et al. [19] found that the topology of COX I phylogenetic tree resembled that of the ITS-based tree, however some *Saprolegnia* isolates clustered apart from those of the same species. Sandoval-Sierra et al. [4] confirmed 29 valid *Saprolegnia* species in 2014, as their and previous studies have indicated several instances of misidentifications and entries with ambiguous information in NCBI GenBank [4,8,13]. The number of undiscovered species is probably much greater than the number of known ones, and new species are still being described today [8,21–23]. At present, the number of accepted species in the genus *Saprolegnia* is a few dozen: 37 according to NCBI Taxonomy [24], while 45 according to the Catalogue of Life [25].

In the present study, we described *Saprolegnia velencensis* sp. n., a novel species from Lake Velence, a natural lake in Hungary. Besides the morphological characterization of vegetative and reproductive structures under different *in vitro* conditions, DNA-level examination and phylogenetic analysis were performed to complete the species description.

## Materials and methods

### Sample collection and isolation

Three water samples were collected at the sailing port of Lake Velence near Velence town, Hungary (47°13’46.5"N 18°39’39.6"E) in August 2021. It is the third largest natural lake in Hungary with 24.5 km^2^ surface area and a shallow average depth (1.4 m). The water temperature is relatively high; in the summer it can reach the 26–28°C. The water has considerably high pH as well (7.8–9.2) and contains a high concentration (1–3 g/L) of dissolved salts (mainly Na- and Mg-hydrogen-carbonates and sulphates) [26,27]. The sampling took place in a public area, and no permission was required for collecting water samples.

Water samples were stored in fridge (+6–8°C) overnight, and transported in cooling bag to the laboratory the next day. Water molds were baited with autoclaved hemp seeds in a 500 mL sterile glass jar, and incubated at room temperature (RT; +20–21°C) until hyphae colonized the seeds (after few days), as described previously by Eszterbauer et al. [28,29]. Then seeds were placed onto glucose–yeast extract agar medium (GY+P+S; pH 6.0), containing 10 g/L glucose, 2.5 g/L yeast extract, 15 g/L agar, 500 mg/L penicillin G sodium salt (P), and 500 mg/L streptomycin sulfate (S). Plates were incubated at RT until hyphae grown the plate. Isolates were maintained on GY+P+S agar, and stored in fridge (+6–8°C). For sub-culturing, a 4-mm agar plug with growing hyphae was cut out and transferred onto a new GY+P+S plate. This procedure was repeated until pure *Saprolegnia* cultures was grown [28,29].

### DNA extraction, PCR amplification, and DNA sequencing

Approximately 10–15 mg of water mold hyphae (wet weight) were collected from GY+P+S agar plate, and placed in a 2 mL tube containing 200 μL double-distilled water (ddH_2_O). DNA was extracted using a Quick-DNA Fungal/Bacterial Miniprep Kit (Zymo Research, USA), following the manufacturer’s protocol with slight modifications. The collected sample were homogenized in the BashingBead tube (containing 750 μL BashingBead buffer) using the TissueLyser LT (Qiagen, Germany) at 50 Hz for 2×5 mins. The rest of the steps were according to the manufacturer’s manual. The quality of extracted gDNA was verified on 1.0% agarose gel, and gDNA concentrations were measured with NanoDrop 2000 spectrophotometer (Thermo Fisher Scientific, USA). Then samples were stored at -20°C. Approximately 710-bp fragment of the region ITS–rDNA (ITS1, 5.8S rDNA and ITS2) was amplified using the universal primers for fungi and fungal-like organisms [30]: ITS1 (5’-TCC GTA GGT GAA CCT GCGG-3’) and ITS4 (5’-TCC TCC GCT TAT TGA TAT GC-3’) described by White et al. [30] with a modified protocol. All PCR assays were performed in Labcycler Basic Thermocycler (SensoQuest, Germany). Thermal cycling conditions were as follows: initial denaturation at 94°C for 5 min, followed by 6 cycles: denaturation at 94°C for 30 s, annealing at 55°C for 30 s, and elongation at 72°C for 60 s, followed by 34 cycles: at 94°C for 30 s, at 52°C for 30 s, and at 72°C for 60 s, with a final elongation step at 72°C for 10 min. PCR was performed in 25 μL volume containing 1×Taq buffer with KCl (Thermo Fisher Scientific, USA), 250 nM of both the forward and reverse primers (IDT, Belgium), 10 mM dNTPs (Sigma, Germany), 1.5 mM MgCl_2_ (Thermo Fisher Scientific, USA) (all final concentrations), 1.25 U recombinant Taq DNA polymerase (Thermo Fisher Scientific, USA), and approximately 10 ng template gDNA.

The approximately 650-bp fragment of gene RNA polymerase II subunit B (RPB2) was amplified using the forward primer SAP-RPB2f (CGA CCG CGA TCA CTA TGG), and the reverse primer SAP-RPB2r (CGA CAC TTC GGC GTC AAT GT) according to Ravasi et al. [18], with a slightly modified protocol. The PCR conditions comprised an initial denaturation (at 95°C for 5 min), followed by 40 cycles: denaturation (at 95°C for 30 s), annealing (at 54°C for 30 s), and elongation (at 72°C for 60s), with a final elongation step at 72°C for 7 min. PCR amplifications were carried out using the same reagents and amounts as described above.

All PCR products were purified using MEGAquick-spin Plus Total Fragment DNA Purification Kit (Intron Biotechnology, Korea). Sanger DNA sequencing was carried out using the BigDye Terminator v3.1 Cycle Sequencing Kit (Life Technologies, USA) according to the manufacturer’s instructions, and detected on Applied Biosystems Genetic Analyzer 3500 (Thermo Fisher Scientific, USA). Similarity search of consensus DNA sequences was performed using the NCBI BLASTn and Megablast feature. For both DNA targets, all consensus sequences were aligned using MAFFT alignment tool with G-INS-i algorithm parameter in Geneious Prime (v2019.2.1.).

### Phylogenetic analysis

Apart from the ITS–rDNA sequences of the novel species (two strains), in total, 62 DNA sequences of relevant *Saprolegnia* spp. obtained from NCBI GenBank were included, such as the reference sequences proposed by Sandoval-Sierra et al. [4] (i.e MOTU sequences). For RPB2 gene, all the 14 *Saprolegnia* spp. sequences (13 submitted by Ravasi et al. [18], one sequence by Jiang et al. [3]) available in GenBank were used as reference sequences for the phylogenetic analyses. Additionally, the ITS–rDNA and RPB2 of seven *Saprolegnia* spp. isolated in our laboratory (four *S*. *ferax*, three *S*. *australis* species identified based on ITS–rDNA sequences) were sequenced and used for phylogeny in the present study (S1 Table).

Maximum likelihood method with tree inference tool RAxML (GTR+G+I model) was applied for the phylogenetic analyses. For tree reconstructions, bootstrap analysis with 1000 repeats was performed. *Achlya caroliniana* was used as outgroup.

### *In vitro* culture

Strains SAP239 and SAP241 maintained on GY+P+S agar medium were sub-cultured under four different *in vitro* conditions at two temperatures (+20–21°C vs. +6–7°C) and at different pH (pH 6.0–7.2 vs. pH 9.0) to increase the chance of obtaining reproductive structures for morphological characterization [17]. Four-mm agar plugs were cut from the plates with sterile biopsy punch, and one plug per well was placed into 24-well plates in 1.5 mL volume of different medium in triplicates. The components of media were (i) sterile distilled water (SDW) only; (ii) sterile, dechlorinated tap water (STW) only; (iii) SDW with fish skin extract (SDW+FS); and (iv) STW with FS (STW+FS). All wells contained antibiotics (500 mg/L penicillin G sodium salt, and 500 mg/L streptomycin sulfate). Mold swabs from each well were regularly examined under light microscope (Zeiss AxioStar Plus, Zeiss). Zoospore production was induced using the method described by Erdei et al. [17]. Colonized agar plugs were incubated at 24-well plates in 1.5 mL GY+P+S broth for three days. Afterwards, agar plugs with hyphal swab were washed in SDW three times, and then incubated in 1.5 mL STW or STW+FS, respectively, for up to two weeks. Zoospore release was monitored under light microscope.

### Nomenclature

The electronic version of this article in Portable Document Format (PDF) in a work with an ISSN or ISBN will represent a published work according to the International Code of Nomenclature for algae, fungi, and plants, and hence the new names contained in the electronic publication of a PLOS ONE article are effectively published under that Code from the electronic edition alone, so there is no longer any need to provide printed copies. In addition, the new species name contained in this work has been submitted to MycoBank from where it will be made available to the Global Names Index. The unique MycoBank number can be resolved and the associated information viewed through any standard web browser by appending the MycoBank number contained in this publication to the prefix https://www.mycobank.org/MB/850128. The online version of this work is archived and available from the following digital repository: Repository of the Library of the Hungarian Academy of Sciences (MTA REAL), http://real.mtak.hu/.

## Results

From the water samples collected, two water mold strains were successfully isolated, and they were named as strain SAP239 and SAP241.

### Molecular characterization of *S*. *velencensis* sp. n. strains

The DNA sequence similarities of strains SAP239 and SAP241 were 100% for ITS–rDNA, and 99.92% for RPB2 gene fragment (S1 and S2 Datasets). The RPB2 consensus sequence of SAP239 shown one SNP at nucleotide positions No. 491 (nucleotide alteration: C/T = Y). The BLAST result of strains SAP239 and SAP241 showed no match with any entries in GenBank. The most closely related ITS sequences to SAP239 and SAP241 were the ones of *S*. *ferax* strains SAP211A (OR004247), SAP213B (OQ236402), SAP233 (OR004248) isolated in our laboratory, with 94.40% identity. The same DNA sequence identity was detected when compared to the DNA sequences in GenBank: *S*. *ferax* strain T1W3-1 (MK911006), *S*. *mixta* (AB219390), *S*. *bulbosa* (AY267011), and *S*. *longicaulis* (AY270032) shown the same (94.40%) sequence identity to the novel strains. In case of RPB2 gene sequences, *S*. *ferax* strain SAP213B (OQ270777) was the most similar to the two strains of *S*. *velencensis* sp. n. (with 95.58 and 95.66% identities, respectively). Surprisingly, *S*. *australis* strain SAP234 (OR004249; with 95.42 and 95.50% identities) had almost as high similarity to the new strains, as *S*. *ferax* strain SAP213B.

### Phylogenetic analysis

**Fig 1:**
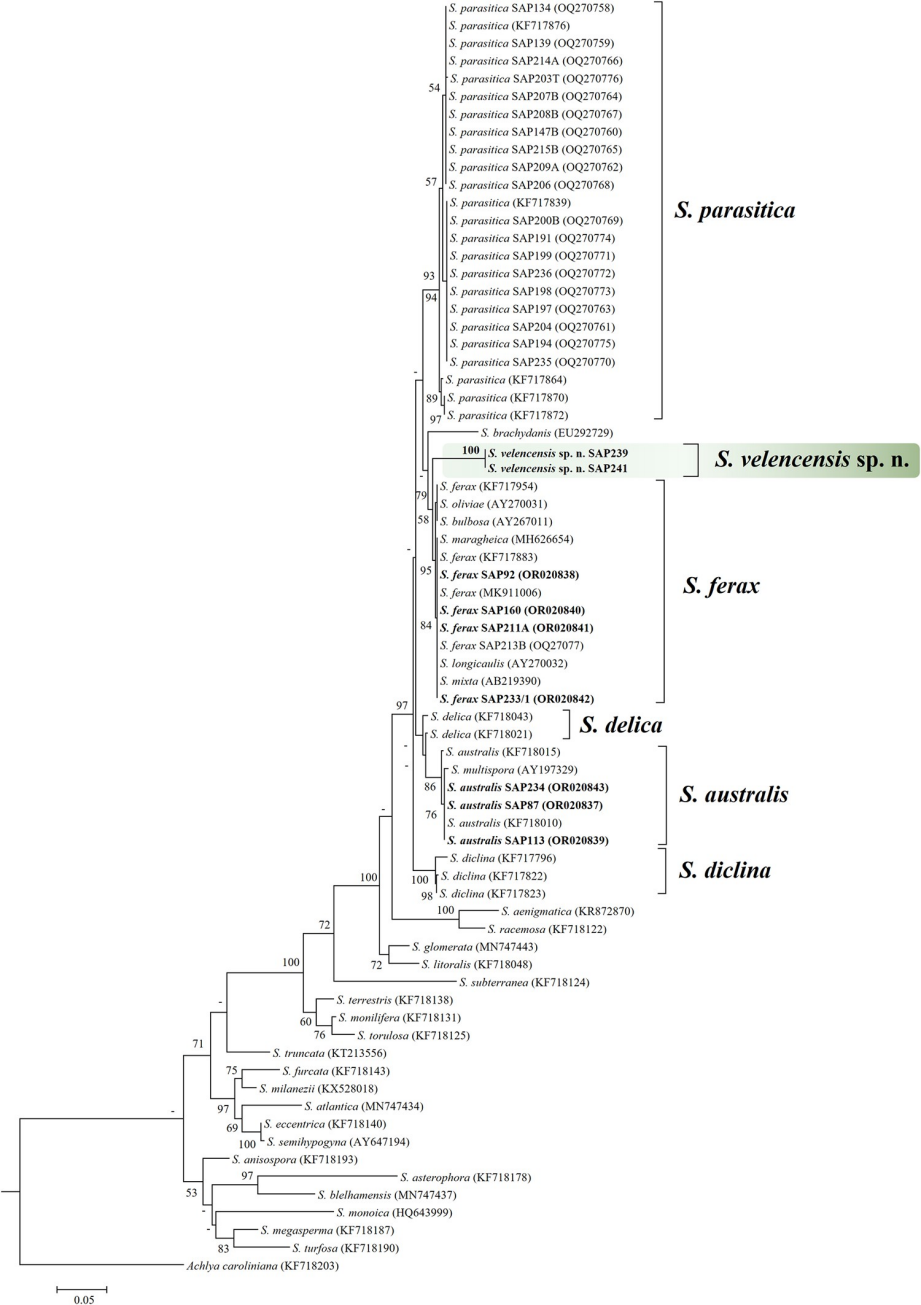
Maximum likelihood tree reconstruction of *S*. *velencensis* sp. n. based on a 730-bp ITS–rDNA alignment. The numbers at the nodes represent the bootstrap support in percentage (>50). The analyses included *Saprolegnia* spp. sequences obtained from the NCBI GenBank. *S*. *australis* and *S*. *ferax* sequences labelled in bold represent samples we isolated previously, but sequenced in the present study. *Achlya caroliniana* was used as an outgroup.

The genetic difference of *S*. *velencensis* sp. n. from other *Saprolegnia* spp. was confirmed with high degree of confidence using RAxML analyses for both ITS–rDNA region and RPB2 gene, based on a 730 and a 611-bp alignment, respectively. The ITS sequences of the *S*. *velencensis* sp. n. strains (SAP239 and SAP241) grouped together with 100% bootstrap support, and their cluster separated well from other *Saprolegnia* spp. (Fig 1). Previously collected sequences of *S*. *ferax* species (SAP92, SAP160, SAP211A, SAP233) branched the most closely to the two strains in the phylogenetic tree. *S*. *longicaulis* (AY270032), *S*. *mixta* (AB219390), *S*. *oliviae* (AY270031), *S*. *maragheica* (MH626654) and *S*. *bulbosa* (AY267011) showed high sequence identities to *S*. *ferax* (99.6–100%), and they located in the *S*. *ferax* cluster with high bootstrap support, up to 95% (Fig 1). Similarly, the ITS–rDNA sequence of *S*. *multispora* (AY197329) was highly similar to *S*. *australis* strains (99.1–99.7% identities), and it grouped with *S*. *australis* strains on the RAxML tree.

**Fig 2:**
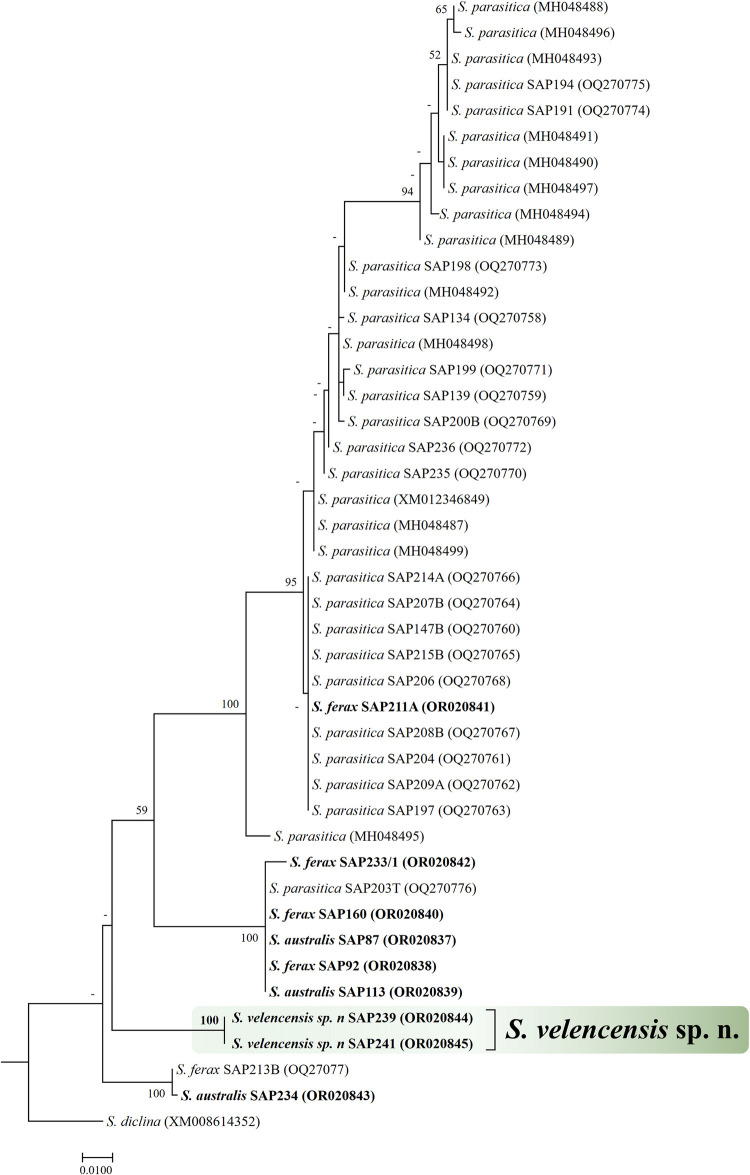
Maximum likelihood tree reconstruction of *S*. *velencensis* sp. n. based on a 611-bp RPB2 gene alignment. The numbers at the nodes represent the bootstrap support in percentage (>50). The analyses included *Saprolegnia* spp. sequences obtained from the GenBank. *S*. *australis* and *S*. *ferax* sequences were obtained from strains we collected previously, but sequenced in the present study (bold).

For RPB2 gene, the topology of RAxML phylogenetic tree was somewhat different from the ITS-based tree regarding some strains (Fig 2). Two strains, one *S*. *ferax* (SAP213B) and one *S*. *australis* (SAP234) clustered more closely to SAP239 and SAP241 than to the rest of *S*. *ferax* and *S*. *australis* strains. However, the two *S*. *velencensis* sp. n. strains clustered together in a separate branch with high bootstrap support (100%). The majority of *S*. *parasitica* sequences and *S*. *diclina* clustered distantly from SAP239 and SAP241 (Fig 2).

**Description of *S*. *velencensis* sp. n.** [urn:lsid:mycobank.org:names:MB850128]

**Holotype:** Lyophilized holotype (strain SAP241) (in agar plugs from *in vitro* culture) was deposited to the Mycology Collection of the Hungarian Natural History Museum (HNHM), Budapest, Hungary under the acc. No.: HNHM-MYC 029995 (coll. No. 112294 BP). Consensus DNA sequences were submitted to NCBI database; holotype SAP241: OR004251 (ITS) and OR020845 (RPB2), paratype SAP239: OR004250 (ITS) OR020844 (RPB2). MycoBank: https://www.mycobank.org/MB/850128.

**Collection site**: at the sailing port on Lake Velence, in town Velence, Hungary (47°13’46.5"N 18°39’39.6"E)

**Date of collection**: 21^st^ August 2021

**Sample type**: Lake water; the strain was isolated from freshwater using hemp seed bait

**Etymology**: The species was named after its collection site at Lake Velence, Hungary.

**Fig 3:**
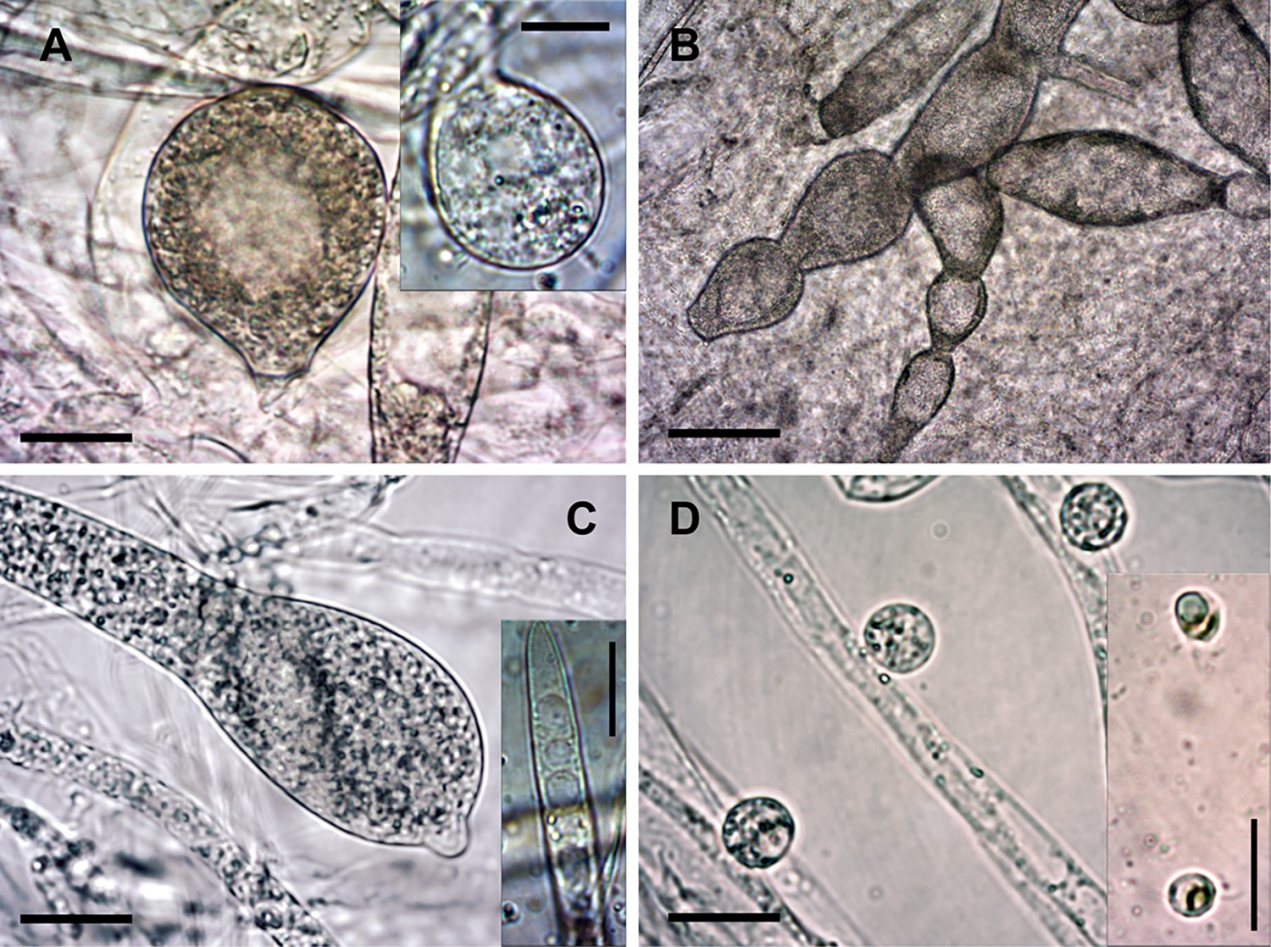
Morphological characteristics of *S*. *velencensis* sp. **n.** A and A inset: Immature oogonia; B: Gemma chains; C: Zoosporangia with papilla; C inset: Mature zoosporangia; D: Secondary cysts; D inset: Zoospores. Scale bars: 20 μm (except for B: 50 μm). Native squash preparations.

**Fig 4:**
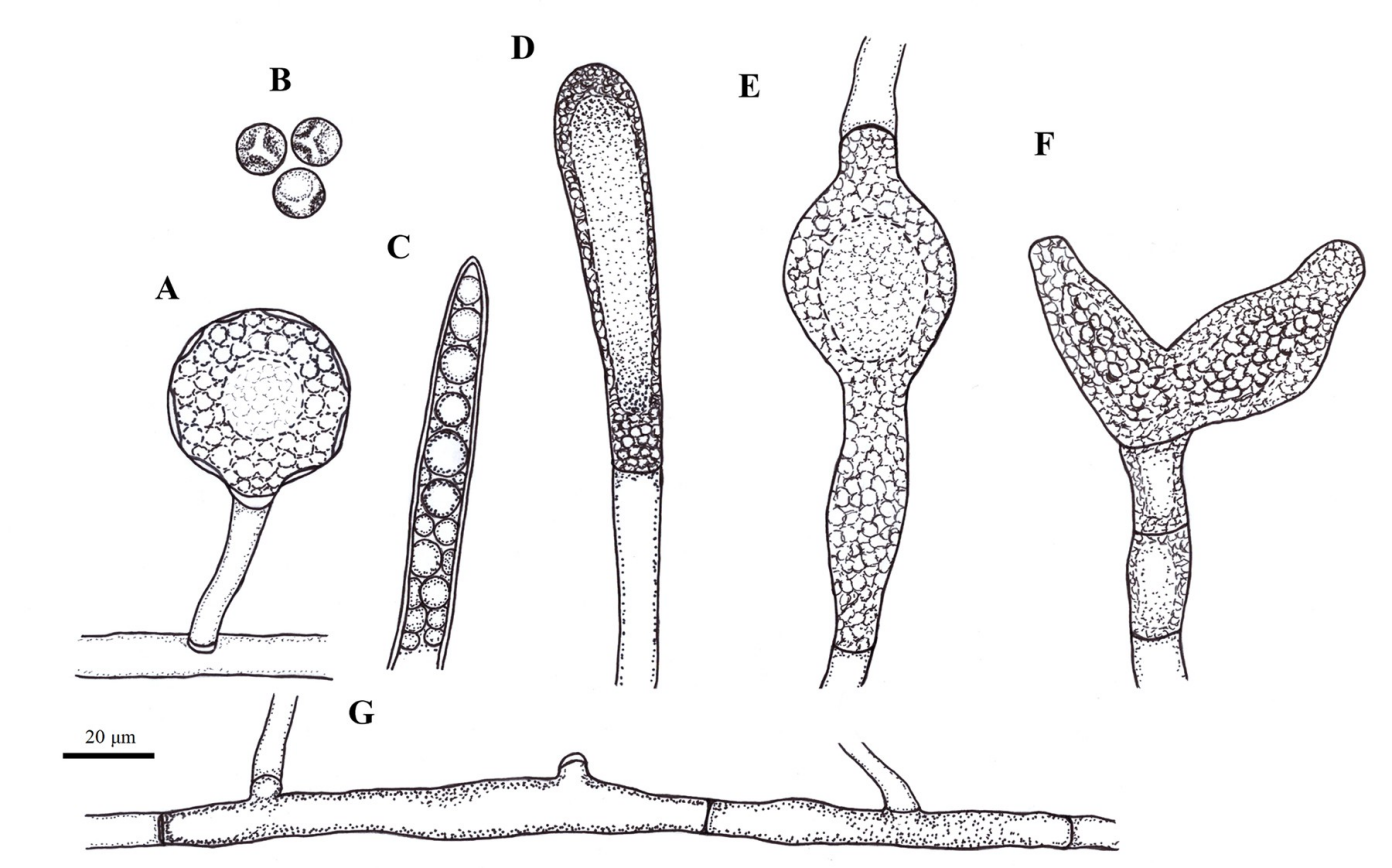
Schematic line drawings of *S*. *velencensis* sp. n. A: Immature oogonia; B: Secondary cysts; C–D: Zoosporangia; E: Intercalary gemma; F: Branched gemma; G: Segmented hypha. Scale bar: 20 μm.

**Morphological characterization**: Hyphae smooth, moderately branched, hyaline, coenocytic, sparsely septate, 13.63±1.74 μm in width (Table 1). Oogonia rare, immature, spherical with slightly pitted wall, and mostly short neck, 40.18±5.93 μm in diameter (29.0–46.73 μm). Gemmae abundant, mostly dense, sometimes branched, fusiform when intercalary and clavate when terminal. Gemmae often form double, sometimes triple or quadruple moniliform chains. Sporangia moderately abundant, clavate, fusiform or navicular, simple to branched, often terminal, sometimes intercalary with an apical papilla prior to zoospore discharge; 163.33±70.07 × 36.69±8.27 μm in size. Secondary zoospores globose, 8.78±0.70 μm in diameter; two flagella. Cysts globose to oval, 11.63±1.77 μm in diameter (Table 1, Figs 3 and 4).

**Table 1:** The measurements of the morphological structures of *Saprolegnia velencensis* sp. n. and other relevant *Saprolegnia* spp. (in μm).

Morphological feature	*S*. *velencensis* sp. n.	*S*. *aenigmatica*	*S*. *anisospora*	*S*. *australis*	*S*. *delica*	*S*. *eccentrica*	*S*. *ferax*	*S parasitica*	*S*. *torulosa*
**Hyphae width**	13.63±1.74	-	-	-	-	-	15−62	18−56	-
**Sporangia length**	163.33±70.07	164.3±59.09	60−227	20−460	-	130−380	60−490 (av. 352)	150−460	80−457
**Sporangia width**	36.69±8.27	21.4±3.4	16−37	15−33	-	25−36	22−78 (av. 51)	20−66	39−44
**Oogonia length**	41.02±14.74	88.6±29.6	-	-	-	-	-	-	-
**Oogonia width**	40.18±5.93	63.4±15.7	40−60	59−80	40−63	30−36	76−99	86−110	50−65
**Zoospore diameter**	8.78±0.70	-	25−33 × 10−12 20−24 × 10−13 11−14 × 8−11	-	10.5−11.5	10−12	-	-	10−12
**Secondary cyst diameter**	11.63±1.77	12.0±0.8	-	10.5−11.7	-	-	7−11 (av. 10)	10−12	-
**References**	Present study	Sandoval-Sierra et al. [22]	Johnson et al. [10]	Johnson et al. [10]	Coker [9]	Johnson et al. [10]	Masigol et al. [11]	Masigol et al. [11]	Johnson et al. [10]

av.: Average.

***In vitro* culture:** Slight changes in the formation and amount of oogonia, gemmae, gemma chains and zoosporangia were observed under the *in vitro* conditions tested (Table 2). The results suggest that room temperature was preferred over the chilled temperature for the formation of vegetative and reproductive structures. The hyphae were slender when incubated in SDW, and they were broader in STW. Most gemmae and gemma chains of 3 to 4 were found in SDW. In the presence of fish skin extract (i.e. at condition SDW+FS and STW+FS), the estimated relative amount of gemmae and zoosporangia was similar when cultured at higher pH (i.e. pH 9), whereas these structures were hardly found in SDW or STW at pH 9 (Table 2). Immature oogonia were observed in the presence of fish skin at RT only. Mature oogonia and antheridia were not seen under any conditions. Intense zoospore release has been detected in STW for up to 14 days after the washing procedure (Table 2).

**Table 2:** Morphological observations in strains SAP241 (holotype) and SAP239 of *S*. *velencensis* sp. n. under different *in vitro* conditions.

Vegetative/ reproductive structures	Room temperature (+21–22°C)								Fridge temperature (+6–7°C)			
	SDW	SDW+FS	STW	STW+FS	SDW pH 9	SDW+FS pH 9	STW pH 9	STW+FS pH 9	SDW	SDW+FS	STW+FS	STW
gemma	+++/+	++/++	++/+	++/+	−/−	+/++	−/−	+/+	+++/+	n.a.	+/+	−/−
gemma chain	+++/+	++/+	−/+	+/−	−/−	+/+	−/−	−/+	+++/+	n.a.	−/+	−/−
zoosporangium	+/−	++/++	−/+	++/+++	−/+	++/+++	−/−	++/+++	−/−	n.a.	−/+	−/−
oogonium	−/−	*+/*+	−/−	*+/*+	−/−	*+/*+	−/−	−/*+	−/−	n.a.	−/−	−/−
zoospore/cyst at Day 1	n.a.	n.a.	+++/−	**−/−**	n.a.	n.a.	n.a.	n.a.	n.a.	n.a.	n.a.	n.a.
zoospore/cyst at Day 14	n.a.	n.a.	+++/−	−/−	n.a.	n.a.	n.a.	n.a.	n.a.	n.a.	n.a.	n.a.

n.a. data not available; SDW: Sterile ddH_2_O; STW: Sterile tap water; FS: Fish skin extract.

*immature.

The relative amount of vegetative and reproductive structures was estimated for up to two weeks of culture. +++: Large number of structures, covering at least half of the microscope field; ++: Large number of structures per field, but countable; +: Few structures per field; −: Structure absent.

**Differential diagnosis:** Based on morphology, *S*. *velencensis* sp. n. could be clearly distinguished from its closest relative, *S*. *ferax*. Sporangia (163.33±70.07 μm in length) were shorter than that of *S*. *ferax* (352 μm in average; based on the characterization by Masigol et al. [11]); the sporangia (36.69±8.27 μm) were also narrower than that of *S*. *ferax* (51 μm in average), however cysts (11.63±1.77 μm in diameter) were somewhat larger than that of *S*. *ferax* (10 μm in average) (Table 1). Compared to other *Saprolegnia* spp., the sporangium length of *S*. *velencensis* (163.33±70.07) was rather similar to that of *S*. *aenigmatica* (164.3±59.09), however the two species differed considerably in other measurements, such as the width of sporangia, the dimensions of zoospores and cysts. The sporangia of *S*. *velencensis* were shorter than that of *S*. *eccentrica*, on the other hand, their width was slightly larger (Table 1). The size of the oogonia (40.18±5.93 μm) of *S*. *velencensis* sp. n. could not be compared with certainty, as only immature oogonia were observed. Nevertheless, they were smaller than the oogonia of other genetically related species (i.e. *S*. *parasitica*, *S*. *ferax*, *S*. *australis*). The oogonia of *S*. *anisospora* (40–60 μm) and *S*. *delica* (40–63 μm) were the most similar in size to that of *S*. *velencensis* sp. n.. The secondary cysts of *S*. *velencensis* possessed similar size to those of *S*. *australis* (Table 1).

## Discussion

In the present study, a novel water mold species, *S*. *velencensis* sp. n. was found, isolated and described. Besides the ITS–rDNA-based molecular identification, the maximum likelihood tree reconstructions of two genomic regions (ITS and RPB2 gene) confirmed that the species examined is indeed new to science. Additionally, the outcome of phylogenetic analyses indicated that the examined DNA sequences of *S*. *longicaulis* (AY270032), *S*. *mixta* (AB219390), *S*. *oliviae* (AY270031), *S*. *maragheica* (MH626654) and *S*. *bulbosa* (AY267011) were identical with those of *S*. *ferax*, while *S*. *multispora* (AY197329) appeared to be identical to *S*. *australis*, which raised the issue of misidentification, as it has been also emphasized in previous studies [4,8,13].

Surprisingly, the topology of the RPB2- and ITS–rDNA-based phylogenetic trees differed in some respects, as certain *Saprolegnia* strains were located at different positions in the RPB2 tree than in the ITS–rDNA tree. This topological variation is a known phenomenon for other organisms [31], and the reason could be the functional difference between coding and non-coding genome parts, as well as the different recombination rate of these genome elements. As the co-presence of multiple water mold species in the same sample/habitat or host is quite likely, certain recombination events between isolates or even species cannot be excluded either, as it is probably the case for other fish parasites, such as myxozoans [32,33].

*S*. *velencensis* sp. n. was isolated from water sample, thus we cannot draw any conclusions regarding its pathogenicity yet. The most common fish pathogen species, *S*. *parasitica*, or the known saprotrophs damaging fish eggs, such as *S*. *australis* and *S*. *ferax* are often found in water samples [7,20,34–36], thus the new species could be still considered potential pathogen. Nevertheless, further *in vivo* experiments are required to understand if *S*. *velencensis* sp. n. has parasitic properties at all. The potential ecological risk of saprolegniosis cannot be excluded either. Previous studies have showed that *S*. *ferax* played a role in the decline of amphibian populations by infecting their eggs [1,5,37,38]. It is also possible that certain unique physico-chemical properties of Lake Velence (i.e. high pH and dissolved salt concentration) may have contributed to the presence of a previously undetected *Saprolegnia* species. Although, some studies have reported that the increased water hardness and sulphate ion concentration correlate negatively with the presence of zoosporic fungi species [37–40]. On the other hand, the results of a recent study on saprolegniosis outbreaks in Egypt have showed that the distribution of *Saprolegnia* spp. was more influenced by water temperature than by salinity [41]. Several studies have suggested that water temperature may be a crucial factor influencing the distribution of water molds [7,11,39,42]. Nam et al. [7] have found that the relative abundance of Saprolegniaceae was higher in the cold season than that in the warm season in South Korea. In addition, Tandel et al. [42] experimentally confirmed that *S*. *australis* could not grow at temperatures below +7°C, whereas *S*. *parasitica* could tolerate a wider range of temperatures (+4–20°C). Meanwhile, other studies have found that decreasing water depth and higher water temperatures favour bacteria which can suppress the growth of water mold, suggesting that water temperature only plays an indirect role in regulating water mold abundance [11,26]. Besides water temperature and salinity, the human factor should also be considered. Lake Velence is a popular tourist spot and recreational area, and the species was found at the sailing port of Velence, where sailing boats are regularly launched and retrieved, thus the introduction of a new water mold species due to human influence cannot be excluded.

*S*. *velencensis* sp. n. did not develop mature oogonia and antheridia under the examined conditions, thus further studies are needed to complete its morphological characterization. Nevertheless, the preliminary *in vitro* examinations suggest that RT is preferred over the colder temperature in the formation of both vegetative and reproductive structures. The temperature tolerance of most *Saprolegnia* species is usually wide [6,37,43], and saprolegniosis may occur both in cold and warm water [2,6], although the most intense hyphal growth has been observed at RT, under *in vitro* conditions [6,43]. Our observation was that immature oogonia were mostly produced in the presence of fish skin extract, in contrast to a previous conclusion suggesting that sexual reproduction of water molds usually occurs under nutrient-depleted conditions [14,44]. Moreover, the thinning of hyphae and the abundance of gemmae and gemma chains in distilled water (SDW) suggest that the new species responded to the nutrient-poor environment by producing persistent vegetative structures. Although the new species shows close phylogenetic relationship with *S*. *ferax*, their morphology differed notably, which further confirmed that the species at hand is indeed new to science.

Similarly to previous studies [2,8,11,39], the description of a new species further emphasizes the need to study water molds in natural waters to obtain a more detailed picture of the species composition of *Saprolegnia* spp. in freshwater habitats.

## Supporting information

S1 Table List of the *Saprolegnia* spp. used for the phylogenetic analyses. (DOCX)

S1 Dataset DNA sequence alignment of the ITS1–rDNA region of *Saprolegnia* spp. examined. (TXT)

S2 Dataset *DNA sequence alignment of RPB2 of* Saprolegnia *spp*. *examined*. (TXT)
