# Supplementary material for: Description of Saprolegnia velencensis sp. n. (Oomycota), a novel water mold species from Lake Velence, Hungary
Source: PLoS One. 2024 Mar 20;19(3):e0298814. doi: 10.1371/journal.pone.0298814 (PMC10954141; doi:10.1371/journal.pone.0298814)
Supplement: S1 Table — (DOCX) [file pone.0298814.s001.docx]

**S1 Table** List of the Saprolegnia spp. used for the phylogenetic analyses.

| **Sample ID** | **Species** | **ITS GenBank Acc. No.** | **RPB2 GenBank Acc. No.** | **Origin** | **Sample type** | **Reference** |
| --- | --- | --- | --- | --- | --- | --- |
| SAP87 | *S. australis* | OR004243 | OR020837 | Dinnyés (Hungary) | fish skin | Present study |
| SAP92 | *S. ferax* | OR004244 | OR020838 | Dinnyés (Hungary) | fish carcass | Present study |
| SAP113 | *S. australis* | OR004245 | OR020839 | Akasztó (Hungary) | fish skin | Present study |
| SAP160 | *S. ferax* | OR004246 | OR020840 | Akasztó (Hungary) | fish skin | Present study |
| SAP211A | *S. ferax* | OR004247 | OR020841 | Dinnyés (Hungary) | water sample | Present study |
| SAP233 | *S. ferax* | OR004248 | OR020842 | Rákos creek (Hungary) | water sample | Present study |
| SAP234 | *S. australis* | OR004249 | OR020843 | Rákos creek (Hungary) | water sample | Present study |
| **SAP239** | *S. velencensis* sp. n. | OR004250 | OR020844 | Lake Velence (Hungary) | water sample | Present study |
| **SAP241** | *S. velencensis* sp.n. | OR004251 | OR020845 | Lake Velence (Hungary) | water sample | Present study |
| SAP134 | *S. parasitica* | OQ236383 | OQ270758 | Gödöllő (Hungary) | water sample | Erdei et al. 2023 |
| SAP139 | *S. parasitica* | OQ236390 | OQ270759 | Gödöllő (Hungary) | water sample | Erdei et al. 2023 |
| SAP147B | *S. parasitica* | OQ236392 | OQ270760 | Akasztó (Hungary) | fish egg | Erdei et al. 2023 |
| SAP191 | *S. parasitica* | OQ236393 | OQ270774 | Lillafüred (Hungary) | fish egg | Erdei et al. 2023 |
| SAP194 | *S. parasitica* | OQ236394 | OQ270775 | Lillafüred (Hungary) | water sample | Erdei et al. 2023 |
| SAP197 | *S. parasitica* | OQ236395 | OQ270763 | Varsád (Hungary) | fish adult | Erdei et al. 2023 |
| SAP198 | *S. parasitica* | OQ236398 | OQ270773 | Varsád (Hungary) | fish adult | Erdei et al. 2023 |
| SAP199 | *S. parasitica* | OQ236399 | OQ270771 | Varsád (Hungary) | fish adult | Erdei et al. 2023 |
| SAP200B | *S. parasitica* | OQ236396 | OQ270769 | Varsád (Hungary) | fish adult | Erdei et al. 2023 |
| SAP203T | *S. parasitica* | OQ236391 | OQ270776 | Dinnyés (Hungary) | fish carcass | Erdei et al. 2023 |
| SAP204 | *S. parasitica* | OQ236400 | OQ270761 | Dinnyés (Hungary) | fish carcass | Erdei et al. 2023 |
| SAP206 | *S. parasitica* | OQ236384 | OQ270768 | Dinnyés (Hungary) | fish adult | Erdei et al. 2023 |
| SAP207B | *S. parasitica* | OQ236389 | OQ270764 | Dinnyés (Hungary) | fish egg | Erdei et al. 2023 |
| SAP208B | *S. parasitica* | OQ236387 | OQ270767 | Dinnyés (Hungary) | fish carcass | Erdei et al. 2023 |
| SAP209A | *S. parasitica* | OQ236388 | OQ270762 | Dinnyés (Hungary) | water sample | Erdei et al. 2023 |
| SAP213B | *S. ferax* | OQ236402 | OQ270777 | Dinnyés (Hungary) | fish egg | Erdei et al. 2023 |
| SAP214A | *S. parasitica* | OQ236386 | OQ270766 | Dinnyés (Hungary) | water sample | Erdei et al. 2023 |
| SAP215B | *S. parasitica* | OQ236385 | OQ270765 | Dinnyés (Hungary) | fish egg | Erdei et al. 2023 |
| SAP235 | *S. parasitica* | OQ236401 | OQ270770 | Gödöllő (Hungary) | fish carcass | Erdei et al. 2023 |
| SAP236 | *S. parasitica* | OQ236397 | OQ270772 | Gödöllő (Hungary) | fish carcass | Erdei et al. 2023 |
|  | *S. aenigmatica* | KR872870 |  | n.a. | n.a. | Sandoval-Sierra et al. 2015 |
|  | *S. bulbosa* | AY267011 |  | Argentina | fish skin | Steciow et al. 2007 |
|  | *S. longicaulis* | AY270032 |  | Argentina | water sample | Steciow 2001 |
|  | *S. mixta* | AB219390 |  | n.a. | n.a. | Inaba. and Tokumasu 2002 |
|  | *S. multispora* | AY197329 |  | United Kingdom | water sample | Paul and Steciow 2004 |
|  | *S. torulosa* | KF718125 |  | Argentina | water sample | Sandoval-Sierra et al. 2014 |
|  | *S. eccentrica* | KF718140 |  | United Kingdom | water sample | Sandoval-Sierra et al. 2014 |
|  | *S. anisospora* | KF718193 |  | Netherlands | water sample | Sandoval-Sierra et al. 2014 |
|  | *S. ferax* | MK911006 |  | Iran | fish skin | Masigol et al. 2020 |
|  | *S. ferax* | KF717883 |  | USA | water sample | Sandoval-Sierra et al. 2014 |
|  | *S. ferax* | KF717954 |  | Ecuador | water sample | Sandoval-Sierra et al. 2014 |
|  | *S. parasitica* | KF717839 |  | United Kingdom | fish adult | Sandoval-Sierra et al. 2014 |
|  | *S. parasitica* | KF717876 |  | Ecuador | water sample | Sandoval-Sierra et al. 2014 |
|  | *S. parasitica* | KF717864 |  | Argentina | water sample | Sandoval-Sierra et al. 2014 |
|  | *S. parasitica* | KF717870 |  | Spain | water sample | Sandoval-Sierra et al. 2014 |
|  | *S. parasitica* | KF717872 |  | Ecuador | water sample | Sandoval-Sierra et al. 2014 |
|  | *S. australis* | KF718010 |  | Spain | water sample | Sandoval-Sierra et al. 2014 |
|  | *S. australis* | KF718015 |  | Ecuador | water sample | Sandoval-Sierra et al. 2014 |
|  | *S. delica* | KF718021 |  | USA | fish adult | Sandoval-Sierra et al. 2014 |
|  | *S. delica* | KF718043 |  | Argentina | water sample | Sandoval-Sierra et al. 2014 |
|  | *S. diclina* | KF717796 |  | Spain | water sample | Sandoval-Sierra et al. 2014 |
|  | *S. diclina* | KF717822 |  | Chile | water sample | Sandoval-Sierra et al. 2014 |
|  | *S. diclina* | KF717823 |  | Chile | water sample | Sandoval-Sierra et al. 2014 |
|  | *S. litoralis* | KF718048 |  | United Kingdom | water sample | Sandoval-Sierra et al. 2014 |
|  | *S. brachydanis* | EU292729 |  | China | fish adult | Ke et al. 2009 |
|  | *S. asterophora* | KF718178 |  | United Kingdom | water sample | Sandoval-Sierra et al. 2014 |
|  | *S. atlantica* | MN747434 |  | Brazil | water sample | Pires-Zottarelli et al. 2022 |
|  | *S. blelhamensis* | MN747437 |  | Brazil | n.a. | Pires-Zottarelli et al. 2022 |
|  | *S. furcata* | KF718143 |  | United Kingdom | water sample | Sandoval-Sierra et al. 2014 |
|  | *S. glomerata* | MN747443 |  | Brazil | n.a. | Pires-Zottarelli et al. 2022 |
|  | *S. maragheica* | MH626654 |  | Iran | n.a. | Chenari Bouket et al. unpublished |
|  | *S. megasperma* | KF718187 |  | Ecuador | water sample | Sandoval-Sierra et al. 2014 |
|  | *S. milanezii* | MN747445 |  | Brazil | n.a. | Pires-Zottarelli et al. unpublished |
|  | *S. monilifera* | KF718131 |  | Ecuador | water sample | Sandoval-Sierra et al. 2014 |
|  | *S. monoica* | HQ643999 |  | n.a. | n.a. | Robideau et al. 2011 |
|  | *S. oliviae* | AY270031 |  | Argentina | water sample | Steciow 2003 |
|  | *S. racemosa* | KF718122 |  | Spain | water sample | Sandoval-Sierra et al. 2014 |
|  | *S. semihypogyna* | AY647194 |  | n.a. | n.a. | Phadee et al. unpublished |
|  | *S. subterranea* | KF718124 |  | USA | water sample | Sandoval-Sierra et al. 2014 |
|  | *S. terrestris* | KF718138 |  | Ecuador | water sample | Sandoval-Sierra et al. 2014 |
|  | *S. turfosa* | KF718190 |  | Germany | water sample | Sandoval-Sierra et al. 2014 |
|  | *S. truncata* | KT213556 |  | Brazil | soil sample | Rocha et al. 2016 |
|  | *Achlya caroliniana* | KF718203 |  | Ecuador | water sample | Sandoval-Sierra et al. 2014 |
|  | *S. parasitica* |  | MH048487 | Switzerland | fish adult | Ravasi et al. 2018 |
|  | *S. parasitica* |  | MH048499 | Switzerland | fish adult | Ravasi et al. 2018 |
|  | *S. parasitica* |  | MH048488 | Switzerland | fish adult | Ravasi et al. 2018 |
|  | *S. parasitica* |  | MH048493 | Switzerland | fish adult | Ravasi et al. 2018 |
|  | *S. parasitica* |  | MH048496 | Switzerland | fish adult | Ravasi et al. 2018 |
|  | *S. parasitica* |  | MH048494 | Switzerland | fish adult | Ravasi et al. 2018 |
|  | *S. parasitica* |  | MH048497 | Switzerland | fish adult | Ravasi et al. 2018 |
|  | *S. parasitica* |  | MH048489 | Switzerland | fish adult | Ravasi et al. 2018 |
|  | *S. parasitica* |  | MH048490 | Switzerland | fish adult | Ravasi et al. 2018 |
|  | *S. parasitica* |  | MH048491 | Switzerland | fish adult | Ravasi et al. 2018 |
|  | *S. parasitica* |  | MH048492 | Switzerland | fish adult | Ravasi et al. 2018 |
|  | *S. parasitica* |  | MH048495 | Switzerland | fish adult | Ravasi et al. 2018 |
|  | *S. parasitica* |  | MH048498 | Switzerland | fish adult | Ravasi et al. 2018 |
|  | *S. parasitica* |  | XM_012346849 | Netherlands | fish fry | Jiang et al. 2013 |
|  | *S. diclina* |  | XM_008614352 | n.a. | n.a. | Jiang et al. 2013 |

**References**

Erdei N, Hardy T, Verebélyi V, Weiperth A, Baska F, Eszterbauer E. New insights into the morphological diversity of *Saprolegnia parasitica* (Oomycota) strains under *in vitro* culture conditions. J Fungi. 2023; 9:982. doi:10.3390/JOF9100982

Inaba S, Tokumasu S. *Saprolegnia semihypogyna* sp. nov., a saprolegniaceous oomycete isolated from soil in Japan. Mycoscience. 2002; 43, 0073–0076 2002. https://doi.org/10.1007/s102670200011

Jiang RHY, de Bruijn I, Haas BJ, Belmonte R, Löbach L, Christie J, et al. Distinctive Expansion of Potential Virulence Genes in the Genome of the Oomycete Fish Pathogen *Saprolegnia parasitica.* PLoS Genet. 2013; 9: e1003272. https://doi.org/10.1371/journal.pgen.1003272

Ke X, Wang J, Gu Z, Li M, Gong X. *Saprolegnia brachydanis*, a new oomycete isolated from zebra fish. Mycopathologia. 2009;167:107-113. doi: 10.1007/s11046-008-9150-z.

Masigol H, Khodaparast SA, Mostowfizadeh-Ghalamfarsa R, Rojas-Jimenez K, Woodhouse JN, Neubauer D, et al. Taxonomical and functional diversity of Saprolegniales in Anzali lagoon, Iran. Aquat Ecol. 2020;54: 323–336. doi:10.1007/s10452-019-09745-w

Paul B, Steciow MM. *Saprolegnia multispora*, a new oomycete isolated from water samples taken in a river in the Burgundian region of France. FEMS Microbiology Letters. 2004; 237: 393–398. https://doi.org/10.1111/j.1574-6968.2004.tb09722.x

Pires-Zottarelli CLA, de Oliveira Da Paixão SC, da Silva Colombo DR, Boro MC, de Jesus AL. *Saprolegnia atlantica* sp. nov. (Oomycota, Saprolegniaceae) from Brazil, and new synonymizations and epitypifications in the genus *Saprolegnia*. Mycol Prog. 2022;21(3), 41. doi:10.1007/s11557-022-01784-2

Ravasi D, De Respinis S, Wahli T. Multilocus sequence typing reveals clonality in *Saprolegnia parasitica* outbreaks. J Fish Dis. 2018;41: 1653–1665. doi:10.1111/JFD.12869

Robideau GP, De Cock AW, Coffey MD, Voglmayr H, Brouwer H, Bala K, Chitty DW, Désaulniers N, Eggertson QA, Gachon CM, Hu CH, Küpper FC, Rintoul TL, Sarhan E, Verstappen EC, Zhang Y, Bonants PJ, Ristaino JB, Lévesque CA. DNA barcoding of oomycetes with cytochrome c oxidase subunit I and internal transcribed spacer. Mol Ecol Resour. 2011;11:1002-1011. doi: 10.1111/j.1755-0998.2011.03041.x. Epub 2011 Jun 20.

Rocha, SCO., Lopez-Lastra, CC, Marano, AV et al. New phylogenetic insights into Saprolegniales (Oomycota, Straminipila) based upon studies of specimens isolated from Brazil and Argentina. Mycol Progress. 2018; 17: 691–700. https://doi.org/10.1007/s11557-018-1381-x

Sandoval-Sierra JV, Diéguez-Uribeondo J. A Comprehensive Protocol for Improving the Description of Saprolegniales (Oomycota): Two Practical Examples (*Saprolegnia aenigmatica* sp. nov. and *Saprolegnia racemosa* sp. nov.). PLoS One. 2015;10: e0132999. doi:10.1371/journal.pone.0132999

Sandoval-Sierra JV, Martín MP, Diéguez-Uribeondo J. Species identification in the genus *Saprolegnia* (Oomycetes): Defining DNA-based molecular operational taxonomic units. Fungal Biol. 2014;118: 559–578. doi:10.1016/j.funbio.2013.10.005

Steciow MM. *Saprolegnia longicaulis* (Saprolegniales, Straminipila), a new species from an Argentine stream, New Zealand Journal of Botany. 2001; 39: 483-488, DOI: 10.1080/0028825X.2001.9512751

Steciow MM. *Saprolegnia oliviae* sp. nov. isolated from an Argentine river (Tierra del Fuego Province, Argentina), FEMS Microbiology Letters. 2003; 219: 253–259. https://doi.org/10.1016/S0378-1097(03)00024-7

Steciow MM, Paul A, Bala K. *Saprolegnia bulbosa* sp. nov. isolated from an Argentine stream: taxonomy and comparison with related species. FEMS Microbiol Lett. 2007; 268:225-230. doi: 10.1111/j.1574-6968.2006.00582.x. PMID: 17328749.
